# Bioinformatics Analysis of Common Genetic and Molecular Traits and Association of Portal Hypertension with Pulmonary Hypertension

**DOI:** 10.1155/2022/9237701

**Published:** 2022-10-20

**Authors:** MingYu Chen, YouPeng Chen

**Affiliations:** The Seventh Affiliated Hospital, Sun Yat-sen University, Shenzhen 518107, China

## Abstract

Portal hypertension (PH) is an important cause of pulmonary arterial hypertension(PAH), but its mechanism is still unclear. We used genetic data analysis to explore the shared genes and molecular mechanisms of PH and PAH. We downloaded the PH and PAH data from the GEO database, and used the weighted gene coexpression network analysis method (WGCNA) to analyze the coexpression modules of idiopathic noncirrhotic portal hypertension (INCPH) and cirrhotic portal hypertension (CPH) and pulmonary hypertension, respectively. Enrichment analysis was performed on the common genes, and differential gene expressions (DEGs) were used for verification. The target genes of INCPH and PAH were obtained by string and cytoscape software, and the miRNAs of target genes were predicted by miRwalk, miRDB, and TargetScan and their biological functions were analyzed; finally, we used PanglaoDB to predict the expression of target genes in cells. In WGCNA, gene modules significantly related to PAH, CPH, and INCPH were identified, and enrichment function analysis showed that the common pathway of PAH and CPH were “P53 signaling pathway,” “synthesis of neutral lipids”; PAH and INCPH are “terminal,” “Maintenance Regulation of Granules,” and “Toxin Transport.” DEGs confirmed the results of WGCNA; the common miRNA functions of PAH and cirrhosis were enriched for “P53 signaling pathway,” “TGF-*β* signaling pathway,” “TNF signaling pathway,” and “fatty acid metabolism,” and the miRNAs-mRNAs network suggested that hsa-miR-22a-3p regulates MDM2 and hsa-miR-34a-5p regulates PRDX4; the target genes of PAH and INCPH are EIF5B, HSPA4, GNL3, RARS, UTP20, HNRNPA2B1, HSP90B1, METAP2, NARS, SACM1L, and their target miRNA function enrichment showed EIF5B, HNRNPA2B1, HSP90B1, METAP2, NARS, SACM1L, and HSPA4 are associated with telomeres and inflammation, panglaoDB showed that target genes are located in endothelial cells, smooth muscle cells, etc. In conclusion, the mechanism of pulmonary hypertension induced by portal hypertension may be related to telomere dysfunction and P53 overactivation, and lipid metabolism and intestinal inflammation are also involved in this process.

## 1. Introduction

Pulmonary arterial hypertension (PAH) is by a variety of causes pathological physiological state of the abnormally elevated pulmonary artery pressure. It can be divided into 5 types: 1) arterial pulmonary hypertension, 2) pulmonary hypertension due to left heart disease, 3) pulmonary hypertension due to pulmonary disease and/or hypoxia, 4) chronic thromboembolic pulmonary hypertension, and 5) pulmonary hypertension due to unknown multifactorial mechanisms. Portopulmonary hypertension (PPHTN) belongs to the first type [1], which is based on portal hypertension, pulmonary arterial hypertension, and pulmonary vascular resistance increased. Studies have pointed out that PPHTN is the three most common types of PAH, and with the continuous improvement of detection methods, the proportion of portal pulmonary hypertension is increasing [2]. About 5%–6% of patients with liver disease have PPHTN, and there is a significant positive correlation between PPHTN and patient mortality [3]. PPHTN is present in 2%–10% of patients with portal hypertension. The pathogenesis of PPHTN is still not completely clear, and splanchnic vasodilatation and portal shunt are considered to be the main causes. Portal hypertension can increase intrahepatic vascular resistance and splanchnic vasodilatation, which leads to increased circulating blood volume and high dynamic state of the body [2, 4]. In addition, portal shunt prevents vasoactive factors from being inactivated by the liver, resulting in imbalance between dilatation factors and contractile factors which ultimately increases pulmonary vascular resistance and eventually leads to pulmonary hypertension [5, 6]. PPHTN is mainly used in drug therapy and liver transplantation, including prostacyclin analogues, endothelin receptor antagonists, and phosphodiesterase-5 inhibitors. Even if, the patients with PPHTN have a high mortality rate, and the best treatment and timing still need to be explored.

With the rapid development of biological genome sequencing, engineering, and molecular biotechnology, researchers could explore and acquire disease-related genetic information so as to explain the pathogenesis of diseases at the gene level and develop effective drugs. To explore the molecular mechanisms and common pathways of portal pulmonary hypertension, therefore, we used the gene expression data and weighted gene coexpression network to analyze PAH and PH-related coexpression gene clusters, explore common functions, and map miRNA and mRNA networks.

## 2. Methods

### 2.1. GEO Datasets

“Portal Hypertension” and “Pulmonary arterial hypertension” were used as keywords to search for Gene expression profiles in NCBI GEO datasets. Inclusion criteria were as follows: 1) Gene expression profile data analyzed by WGCNA should be greater than 15 to ensure accuracy; 2) gene expression profile data should include experimental and control groups; and 3) original data should be available. Finally, GSE77627 and GSE113439 were selected for WGCNA analysis, and GSE77627 and GSE53408 were selected for GEO2R analysis.

### 2.2. WGCNA Analysis

The data of GSE77627 are divided into cirrhotic portal hypertension (CPH) and idiopathic noncirrhotic portal hypertension (INCPH). Firstly, the top 25% genes were selected according to their variance (CPH and PH took the top 5000 genes). The matrix was transposed and converted into a matrix with sample rows and genes in columns for WGCNA analysis (using the WGCNA software package in R.4.0.5.). The Hclust function of the R language was used for hierarchical clustering analysis to exclude outlier samples. Then, according to the standard of the scale-free network, the “pickSoftThreshold” function in WGCNA software package is used to select the appropriate soft threshold *β* (ranging from 1 to 20). Next, soft threshold beta was calculated using Pearson's analysis, and the correlation between genes and gene matrix to construct adjacency matrix, calculated by the formula: *a*_*ij*_ = | *S*_*ij*_ | beta (*a*_*ij*_: adjacency matrix genes between *i* and *j*, *S*_*ij*_: consisting of all genes of Pearson's correlation coefficient of similarity matrix, beta: soft threshold). Then, the adjacency matrix transforms the topological overlap matrix (TOM) and the corresponding dissimilarity (1−TOM). The hierarchical clustering tree was further constructed to divide the similar gene expression into different modules. Finally, the expression profile of each module was summarized using a modular characteristic gene (ME), and the correlation between ME and clinical features was calculated. Modules with high correlation coefficients with clinical features were analyzed (*p* < 0.001), and genes in these modules were selected for subsequent analysis. In this study, scale independence was set at 0.85, and the soft threshold *β* in WGCNA analysis of PAH was the value estimated by WGCNA (PAH = 5, LCPH = 16, INCPH = 12, PH = 20). The other parameters are: network Type = “unsigned”, min Module Size = 30, merge Cut Height = 0.25.

### 2.3. The Genes and Shared Genes of Screened PAH and PH Modules Were Identified

We selected gene modules that were significantly positively associated with the trait (*p* < 0.001) and selected genes in the correlation module based on GS and MM values (ABS (GS) > .2& ABS (datKME$MM. Module) > .8). At the same time, the genes with significant correlation between PH and PAH groups were overlapped by Venn diagram to find the shared genes. Finally, Cytoscape's ClueGo function was used for GO enrichment analysis and biological analysis of shared genes.

### 2.4. Shared PAH and PH Genes Were Analyzed by DEGs

We used DEGs to analyze GSE77627 and GSE53408 databases. Using GEO2R of PubMed to analyze GSE77627 and GSE53408 databases online, respectively, and then filtered the data in R language, respectively, including: 1. Adjusted the logFC threshold. 2. Adjusted adj. P threshold. 3. Gene filtering without specific meaning (such as loC-beginning genes and Mir-/MT-beginning genes). 4. Deleted data with both high and low expression. GO functional enrichment analysis and biological analysis were performed for high and low expression of PAH and PH. In DEGs, we still divided GSE77627 data into two groups for GEO2R analysis, that is, CPH compared with the normal group (G1), INCPH compared with the normal group (G2), and PH compared with the normal group (G3). Finally, the differential gene analysis with PAH was performed in G1, G2, and G3 groups, respectively (fC. Cutoff < −1.8, FDR. Cutoff < −0.5). Cytoscape's ClueGo function was used for GO functional enrichment analysis and biological analysis of shared genes.

### 2.5. Identification of miRNA in Shared PH and CPH

miRNA is a noncoding RNA that can inhibit mRNA expression and degrade mRNA to regulate gene expression, which is of key significance for the occurrence and development of diseases. Therefore, we explored the regulation of comiRNA on PH and CPH. The upregulated miRNAs of PAH were searched in the Human miRNA Disease Database (HMDD). Since the HMDD did not record miRNA of PH, CPH is a late complication of cirrhosis. So the miRNAs of cirrhosis were used to replace CPH, and the shared miRNAs between them were identified, and their biological functions and target genes were predicted in miRpath. Finally, the target genes of miRpath, the common genes of WGCNA, and DEGs were screened, and the miRNA-mRNA network map was drawn.

### 2.6. Identification of miRNAs' Function in Shared PH and INCPH

String software was used to analyze the shared downregulated genes of WGCNA and DGEs in PH and INCPH, and then Cytoscape was used to identify target genes. Finally, miRNA prediction of target genes was performed by miRwalk, TargetScan, and miRDB. Its biological function was predicted using miEAA 2.0 [7] and miRpath.

### 2.7. Expression of Target Genes in Cells

PanglaoDB software(PanglaoDB - A Single Cell Sequencing Resource For Gene Expression Data), a database that analyzes the expression of genes in those cell subsets, was used to predict the expression of target genes in cells.

## 3. Results

### 3.1. GEO Information

**Figure 1:**
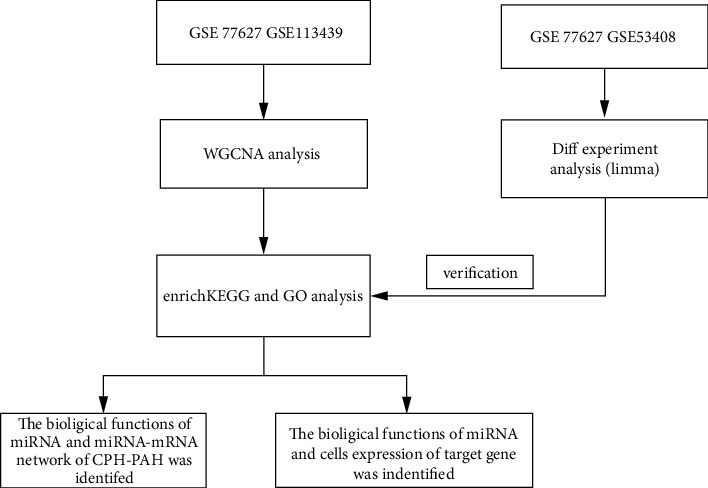
Study workflow.

Figure 1 shows the workflow of the present study. Transcriptome data of GSE113439 and GSE77627 were used for WGCNA analysis, and transcriptome data of GSE77627 and GSE53408 were used for DEGs.

### 3.2. Analysis Results in WGCNA

**Figure 2:**
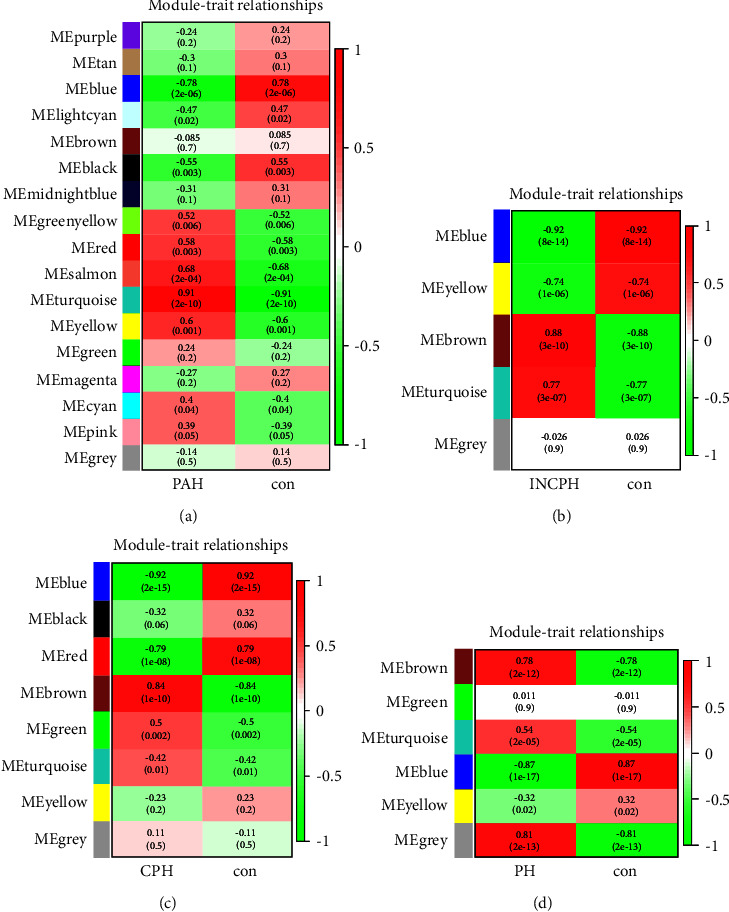
Weighted gene coexpression network analysis (WGCNA). (a) Module–trait relationships in PAH. Each cell contains the corresponding correlation and *p*-value. (b) Module–trait relationships in INCPH. Each cell contains the corresponding correlation and *p*-value. (c) Module–trait relationships in CPH. Each cell contains the corresponding correlation and *p*-value. (d) Module–trait relationships in PH. Each cell contains the corresponding correlation and *p*-value. PAH,pulmonary arterial hypertension; INCPH, idiopathic noncirrhotic portal hypertension; CPH,cirrhotic portal hypertension.

In WGCNA, GSE113439 was divided into 17 modules in total, among which two modules (Salmon, Turquoise) were significantly positively correlated with PAH, including 7 and 1982 screened genes according to GS value and MM value. One module (blue) was negatively correlated with the PAH group, including 551 screened genes (Figure 2(a)). There were 8 modules in CPH, among which one module (Brown) was significantly positively correlated with CPH, including 526 genes after screening. Two modules (blue and red) were negatively correlated with CPH, including 678 and 85 genes (Figure 2(b)). INCPH has a total of 5 modules, of which 2 modules (Brown, Turquoise) were positively correlated with INCPH, including 461 and 988 genes after screening. Two modules (yellow and blue) were negatively correlated with INCPH, and the screened genes included 452 and 1183 genes (Figure 2(c)). PH has a total of 6 modules, of which 2 modules (Brown, Turquoise) were positively correlated with INCPH, including 340 and 473 genes after screening.

**Figure 3:**
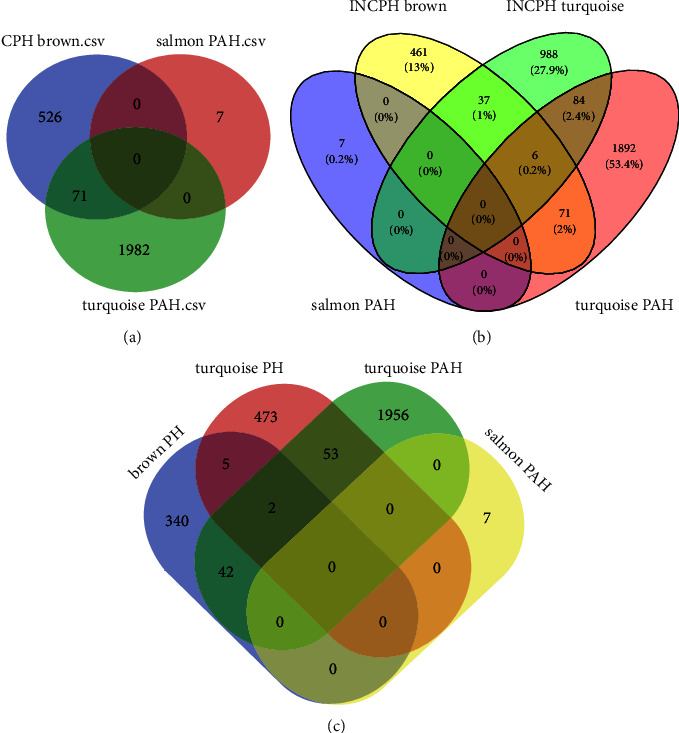
(a) The shared genes between the salmon and turquoise modules of PAH and brown module of CPH by overlapping them.(b) The shared genes between the salmon and turquoise modules of PAH and brown and turquoise module of CPH by overlapping them. (c) The shared genes between the salmon and turquoise modules of PAH and brown and turquoise module of PH by overlapping them. PAH, pulmonary arterial hypertension; INCPH, idiopathic noncirrhotic portal hypertension; CPH, cirrhotic portal hypertension.

**Figure 4:**
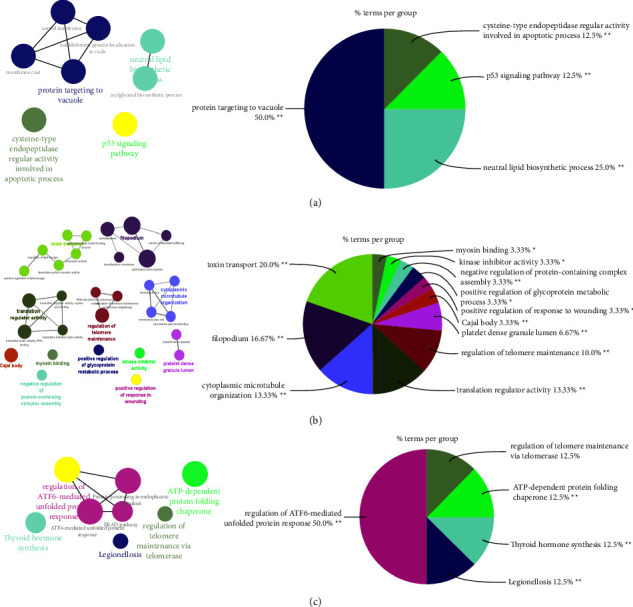
ClueGO enrichment analysis. (a) CPH-PAH:The interaction network of GO terms generated by the cytoscape plug-in ClueGO. The significant term of each group is highlighted. Proportion of each GO terms group in the total. (b) INCPH-PAH: The interaction network of GO terms generated by the cytoscape plug-in ClueGO. The significant term of each group is highlighted. Proportion of each GO terms group in the total. (C) PH-PAH: The interaction network of GO terms generated by the cytoscape plug-in ClueGO. The significant term of each group is highlighted. Proportion of each GO terms group in the totalGO, gene ontology. ^*∗∗*^*p* < 0.05. PAH, pulmonary arterial hypertension; INCPH, idiopathic noncirrhotic portal hypertension; CPH, cirrhotic portal hypertension; PH, portal hypertension.

In CPH and PAH positive correlation module, there were 71 common genes (Figure 3(a)), GO analysis included “protein targeting to vacuole” “P53 signaling pathway,” “neutral lipid biosynthesis,” and “cysteine-type endonuclease regulator activity involve in apoptotic process” (Figure 3(a)). There were 161 common genes in the positive correlation module of INCPH and PAH (Figure 3(b)), and the enrichment analysis was “telomere maintenance regulation,” “translation regulator activity,” and “toxin transport” (Figure 4(b)). There were 97 common genes in the positive correlation module of PH and PAH (Figure 3(c)), and the enrichment analysis were “telomere maintenance regulation” and “ATF-6 mediated unfold protein response” (Figure 4(c)).

### 3.3. The Biological Characteristics of Shared PAH and PH Genes Were Analyzed Using DEGs

**Figure 5:**
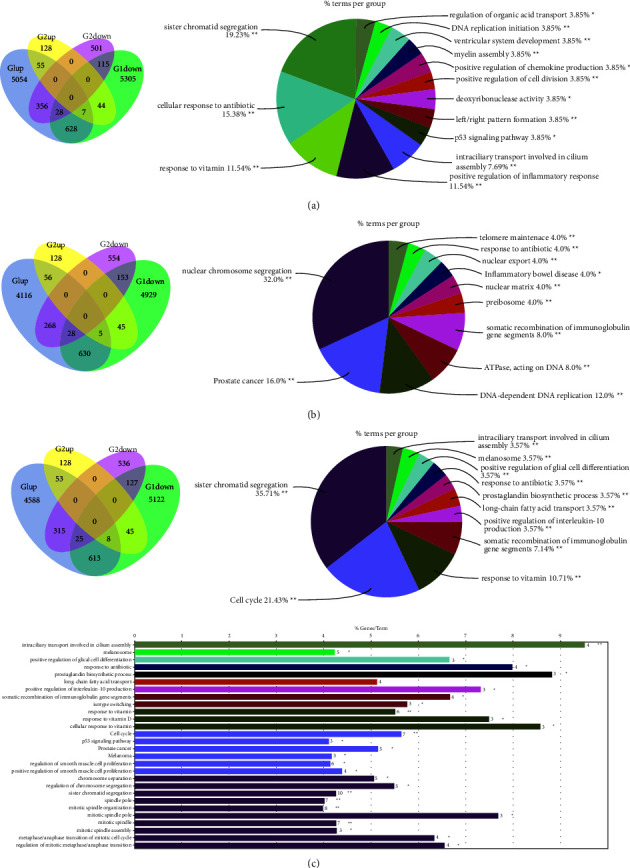
Identification of the common DEGs and ClueGO enrichment analysis. (a) The Venn diagram of the downregulated genes in PAH and CPH, the interaction network of GO terms generated by the cytoscape plug-in ClueGO. (b) The Venn diagram of the downregulated genes in PAH and INCPH, the interaction network of GO terms generated by the cytoscape plug-in ClueGO. (c) The Venn diagram of the downregulated genes in PAH and PH, the interaction network of GO terms generated by the cytoscape plug-in ClueGO. PAH, pulmonary arterial hypertension; INCPH, idiopathic noncirrhotic portal hypertension; CPH, cirrhotic portal hypertension. PH, portal hypertension. GO, gene ontology. ^*∗∗*^*p* < 0.05.

We also analyzed GSE77627 and GSE53408 data by DEGs, and the results showed that CPH and PAH had 146 downregulated common genes (supplementary materials). GO analysis showed “Positive regulation of the inflammatory response”, “Sister chromatid separation,” and “P53 signaling pathway”(Figure 5(a)). There were 181 downregulated genes in INCPH and PAH (supplementary materials), and GO analysis showed “nuclear chromosome segregation,” “inflammatory bowel disease,” and “telomere maintenance” (Figure 5(b)). There were 152 downregulated genes in PH and PAH (supplementary materials), and GO analysis showed “sister chromosome segregation” and “cell cycle (P53 signaling pathway)” (Figure 5(c)).

### 3.4. Identification of miRNAs in Shared PH and CPH

Two miRNAs were upregulated (hsa-mir-22-3p, hsa-mir-23b-3p) and two were downregulated (hsa-mir-146A-5p, hsa-mir-34a-5p). miRpath showed “fatty acid metabolism,” “fatty acid synthesis,” “endoplasmic reticulum protein processing,” “P53 signaling pathway,” “TGF-*β* signaling pathway,” and “TNF signaling pathway.”

### 3.5. miRNA-mRNA Network of PH and CPH

The common gene of the target gene of upregulated miRpath, the shared gene of WGCNA and downregulated DEGs were screened, and there was a total of one gene, MDM2, which was regulated by has-miR-22a-3p. The common gene of the target gene of downregulated miRpath, the shared gene of WGCNA, and upregulated DEGs were screened, and there was one gene in total, PRDX4, which was regulated by has-miR-34a-5p.

### 3.6. miRNA's Function in PH and INCPH

MiRwalk, TargetScan, and miRDB were performed on the screened target genes, respectively, for miRNA intersection (supplementary materials). Among them, miAEE showed that miRNA of EIF5B, HNRNPA2B1, HSP90B1, METAP2, NARS, SACM1L, and HSPA4 were related to telomere function. In addition, we selected the genes that derived from the intersection of target genes with genes enriched for telomeres by WGCNA and DEGs, and miRpath showed that they were related to cell circle, P53, and DNA repair.

### 3.7. Expression of Target Genes in Cells

MDM2 is expressed in hepatocytes, fibroblasts, and adipocytes in basal cell size; PRDX4 is expressed in hepatic stellate cells, fibroblasts, endothelial cells, and adipocytes. EIF5B, HNRNPA2B1, HSP90B1 METAP2, NARS, SACM1L, HSPA4, GNL3, UTP20, and RARS are expressed in endothelial cells, smooth muscle cells, and fibroblasts.

## 4. Discussion

Through WGCNA, DEGs analysis, and miRNA identification, it was concluded that the common pathways of CPH and PAH were “lipid (accumulation) metabolism,” “inflammatory response,” and “P53 signaling pathway.” The common pathways of INCPH and PAH were “telomere,” “intestinal inflammation,” and “toxin transport.” The common pathways of PH (including CPH and INCPH) and PAH included the P53 signaling pathway and telomere.

Liver cirrhosis is the precondition for CPH, and the development of HC is inseparable from the activation of hepatic stellate cells (HSCs). The phenotype changes from static to myofibroblasts with contraction and secretion of extracellular matrix, causing liver fibrosis. The formation of portal hypertension is also related to liver fibrosis and the contractility phenotype of HSCs. In addition, portal hypertension is closely related to hepatic sinusoidal endothelial cells and platelets/thrombocyte [8]. INCPH is a clinically pathological disease with the clinical features of portal hypertension after the exclusion of cirrhosis and known risk factors. However, with further study, portal sinus vascular disease was proposed, and the definition of INCPH was broadened to include the prephase of INCPH and the PH of known liver disease that are concomitant [9, 10].

The liver is involved in fat metabolism, including triglyceride metabolism, production, and transportation of very low density lipoprotein. Neutral lipids are inert lipids whose synthesis is the first step of lipid droplet (LD) formation [11]. LD is an important dynamic organelle for lipid storage and plays a major role in cell metabolism regulation, coordinating lipid and protein metabolism in cells. Excessive neutral lipid synthesis can also affect lipid droplet metabolism and the function of lipid-associated proteins. However, the relationship between lipid droplets and inflammation is not simple, because lipid droplets can store inflammatory lipid mediators such as arachidonic acid and contain mechanisms for the synthesis of eicosanoic acid. Therefore, lipid droplets are considered a special point for the synthesis of eicosanoic acid in inflammatory responses [12–14]. HSCs also contain LD and liver cell lipid drops, in addition to the neutral lipid and stored retinoic ester/vitamin A. The activation of HSCs is the first step in liver injury and repair. HSCs' activation into muscle fibroblasts plays the role of pathogenic factors, along with the change of LD and deposition of extracellular matrix. Some scholars have described in the process of HSCs activation, LD is a dynamic process accompanied by a reduction in retinol and the synthesis of large amounts of neutral lipids from polyunsaturated acid (exogenous arachidonic acid) [15, 16]. It has also been suggested that triglycerides play a key role in HSCs activation [17]. The role of oxidative stress has been reported in various types of liver disease, liver fibrosis, and cirrhosis [18]. When various etiologies lead to an imbalance between oxidation and antioxidants, both free radicals and oxidative stress products can activate HSCs and eventually lead to liver fibrosis [19, 20]. Inflammation promotes the occurrence and development of liver disease. When the liver is damaged and liver cells die, inflammation is promoted to protect the liver by eliminating pathogens. However, once the inflammation is maladjusted or transformed into chronic inflammation, liver fibrosis will be promoted. Hepatocytes, HSCs, Kupffer cells, platelets, endothelial cells, macrophages, NK cells, and lymphocytes are all involved in inflammatory responses [21]. Inflammatory cells generate soluble medium such as arachidonic acid metabolites, chemokines, and cytokines induce inflammatory factors to the injury, oxidative stress, and inflammation in the liver disease development to play the role of a mutual promotion, oxidative stress can through signal transduction pathways (including P53) promote the expression of proinflammatory genes that cause inflammation, inflammation further enhances oxidative stress by releasing more free radicals [22]. CPH is a consequence of liver cirrhosis and is associated with increased intrahepatic resistance and visceral blood flow. Increased intrahepatic resistance is associated with structural malformations caused by liver cirrhosis and arachidonic acid metabolites [23]. P53 is a well-known tumor suppressor gene. With further research, P53 has been found to play an important role in oxidative stress, metabolism, and the cell cycle of the liver, such as in metabolic syndrome, nonalcoholic liver disease (inflammation), hepatic insulin resistance, and liver regeneration, in addition to regulating DNA and cell stagnation in cancer [24]. Some scholars have found that the expression level of P53 in chronic hepatitis B cirrhosis is higher than that in chronic hepatitis B and normal controls [25]. Some studies have shown that P53 plays a role in weakening fibrosis [26]. Interestingly, other studies have revealed that P53 induces the expression of CTGF and promotes liver fibrosis and suggested that the P53/CTGF pathway may be a target for the treatment of liver fibrosis [27]. P53 also plays an important role in pulmonary hypertension. Studies had shown that the inactivation of P53 in rats induces the formation of pulmonary hypertension and vascular remodeling [28, 29]. Another study had shown that in mice with hypoxia-induced pulmonary hypertension and idiopathic PAH, the expression of P53 in lung smooth muscle cells decreased and the expression of P53 in lung endotheliocytes and hypoxia-inducible factor-2*α* (HIF-2*α*) increased. Pulmonary hypertension is due to vascular remodeling caused by increased proliferation of pulmonary smooth muscle cells and apoptosis of pulmonary endothelial cells, resulting in endothelial cell dysfunction and continuous vasoconstriction [30]. HIF-2 *α* induces endothelial cell transformation into mesenchymal cells leading to severe pulmonary hypertension [31]. Endothelial cells Kruppel-like factor 2 (KLF2) mediates endothelium-dependent vascular homeostasis by regulating endothelial genes, which can induce the formation of endothelium-dependent nitric oxide and inhibit the expression of endothelin-1. However, overexpressed P53 promotes vasoconstriction and thrombosis by inhibiting this factor, leading to reduced endothelial cell-dependent nitric oxide release and NO utilization, which is independent of the NF-*κ*B pathway [32]. P53 can promote inflammation and damage endothelial function by inhibiting KLF2, and can also achieve anti-inflammatory processes by inhibiting NF-*κ*B [33]. Surprisingly, P53 and the ER DHRS3 gene contribute to the formation of lipid droplets [34]. In the results of our experiment, the downregulated genes reflecting the P53 pathway were CCNB2, CCNE2, and MDM2, which downregulate the expression of P53. Therefore, our results show that the upregulated expression of the P53 pathway is the common pathway of CPH and PAH. The common CPH and PAH genes were downregulated by MDM2 and upregulated by PRDX4. MDM2 is a negative stabilizing factor for P53. Under normal physiological conditions, MDM2 can degrade the P53 proteasome to stabilize it at a low level, but under harmful stimulation, the reduced binding of MDM2 to P53 can promote the high level of P53 [35]. PRDX4 is a peroxidase that scavenges free radicals, but overexpression leads to cell proliferation [36, 37]. Some scholars have stated that overexpression of PRDX4 does not show antioxidation, but induces inflammation and aggravates the development of pulmonary fibrosis [38]. We hypothesized that the interaction of overexpression of P53, oxidative stress, and inflammation leads to pulmonary hypertension caused by portal hypertension in cirrhosis.

The common pathways of INCPH and PAH are telomeres, (intestinal) inflammation, and toxin transport. Maintenance of telomeres ensures the correct replication of genetic material and protects DNA from damage to ensure genomic stability and accuracy. Telomere dysfunction can lead to cell cycle arrest, aging, and genomic instability, and is associated with bone marrow failure syndrome and cancer [39]. Studies have shown that telomere dysfunction leads to hepatic endothelial dysfunction and necrosis of perivascular hepatocytes, leading to nodular hyperplasia and INCPH, and the authors state that this disorder contributes to pulmonary vascular malformation and hypoxia [40]. What is clear is that short telomeres are significantly associated with pulmonary fibrosis [41]. Damage to lung epithelial cells increases myofibroblast resistance to apoptosis, leading to accumulation of extracellular matrix and the formation of pulmonary fibrosis and ultimately resulting in pulmonary hypertension ultimately [42, 43]. In the presence of portal hypertension, dilation of visceral vessels and congestion of neovascularization lead to impaired intestinal barrier function, and in recent years, the proposed enterohepatic axis has clarified the relationship between the gut and the liver, including the mechanisms of inflammation, dysbiosis, increased intestinal permeability, and endotoxemia. PH leads to an increase in intestinal permeability and when intestinal inflammation occurs, bacteria will enter the circulatory system and continuously activate the immune system, resulting in chronic inflammation and visceral dysfunction [44]. Interestingly, studies have shown that pulmonary hypertension is associated with increased intestinal permeability and circulating endotoxins, and the intestine-lung association in PAH has been proposed [45]. In addition, intestinal microbiome disorders are a potential cause of PAH [46]. Bone morphogenetic protein receptor type II (BMPR-II) mutations are the basis of most hereditary PAHs, but only 20-30% of hereditary PAHs develop. Studies have indicated that inflammation or endotoxin is a trigger point for BMPR-II to cause PAH and have demonstrated that the deficiency of BMPR-II exhibits a proinflammatory phenotype that leads to pulmonary vascular remodeling [47]. Moreover, the presence of portal hypertension causes endotoxins released from the intestine into the visceral venous circulation to enter the pulmonary circulation directly without passing through the liver for catabolism. Telomere dysfunction can cause tissue inflammation. One study demonstrated that in telomere dysfunction, activation of the atM-YAP1-pro-IL-18 pathway in intestinal epithelial cells is a key cause of intestinal inflammation [48]. Telomere dysfunction results in DNA damage and P53 activation, resulting in cell growth stagnation, senescence, and apoptosis. Meanwhile, telomere dysfunction can deeply inhibit peroxisome proliferator-activated receptor activators 1*α* and 1*β* (PGC-1*α* and PGC-1*β*) by inducing P53 activation, ultimately leading to mitochondrial dysfunction and promoting cell senescence. With the deepening of the research on PAH, scholars found that cell aging phenotype in irreversible PAH plays a decisive role in apoptosis and senescence, TNF-*α* induced pulmonary cells to the aging phenotype lead to vascular dysfunction and proved that the aging of vascular smooth muscle cell proliferation of cells can be induced in vitro [49]. Telomere dysfunction is also closely associated with cellular REDOX reactions and cellular metabolism [50]. GNL3 and HNRNPA2/B1 are derived from the intersection of ten target genes with genes enriched at telomeres by WGCNA and DEGs. The full name of GNL3 is G protein nucleolar 3. It is also known as NS. Some studies have shown that GNL3 (NS) reduces the formation of telomere dysfunction by shortening the dynamic association of telomeric repeat-binding factor 1(TRF1) and telomeres [51]. In addition, some researchers show that NS can also promote the degradation of TRF1 and prolong the telomere protecting cells that are dividing from the short telomere [52]. HNRNPA2B1 is an RNA binding protein (RBP), which is involved in telomere regulation and genome integrity. HnRNP can participate in the regulation of telomere activity through a variety of steps, such as controlling the location of telomere repeat RNA, regulating the connection between telomere complex components including telomere DNA binding to telomerase reverse transcriptase and expression of telomerase regulators, and transcriptional or post-transcriptional regulation of telomerase regulators, and so on [53]. Therefore, we proposed the hypothesis that portal hypertension can lead to oxidative stress, changes in intestinal wall structure, bacterial translocation, intestinal lipid peroxidation, sepsis, etc [54]. Under the action of the hepatointestinal axis and the enteropulmonary axis, intestinal inflammation, imbalance of endotoxin, and microflora lead to the formation of pulmonary hypertension; meanwhile, oxidative stress imbalance and inflammation can also lead to telomerase inactivation and telomere shortening, which promote the occurrence of PAH [55].

We also analyzed PH (including CPH and INCPH) and PAH by DEGs and WGCNA. Their common pathways include the P53 signaling pathway or telomere, and we thought of it as a potential mechanism. Portal hypertension can lead to oxidative stress, bacterial translocation, intestinal lipid peroxidation, sepsis, etc. Oxidative stress and inflammation will lead to telomere dysfunction and then induce the activation of P53, while overactivated P53 and telomere dysfunction can also induce oxidative stress and inflammation, forming a vicious cycle that promotes senescence and dysfunction of pulmonary vascular cells, reduces the secretion of NO, causes vascular dilatation dysfunction, and promotes or accelerates the formation of pulmonary hypertension.

**Figure 6:**
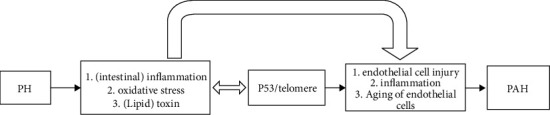
The disease road model. P53 and/or telomere plays an important role in the disease road model. PH can lead to oxidative stress, (intestinal) inflammation and (lipid) toxin.Oxidative stress and inflammation will lead to telomere dysfunction and then induce the activation of P53, while overactivated P53 and telomere dysfunction can also induce oxidative stress and inflammation, forming a vicious cycle that promotes endothelial cell injury, inflammation, and aging of endothelial cells, and promote or accelerate the formation of PAH.PAH, pulmonary arterial hypertension; PH, portal hypertension.

Portal pulmonary hypertension is a rare complication of portal hypertension. At present, there are few studies on the mechanism of the disease. This is the first study to use the WGCNA method to construct a coexpression network to explore the relationship between PH (including INCPH and CPH) and pulmonary hypertension. Our findings reveal several key genes and a common pathway that play an important role in the etiology of portal pulmonary hypertension, which may enhance our understanding of the molecular mechanisms of this disease. In this study, we believe that P53 signaling pathway/telomere dysfunction may be the pathogenesis of the disease, and the mechanism (Figure 6) may be that oxidative stress and inflammation lead to telomere dysfunction, induce P53 activation, telomere dysfunction, and excessive activation of P53 induces oxidative stress, inflammation, etc., promoting pulmonary vascular cell aging and dysfunction, and reduced NO secretion leads to vasodilation dysfunction, which leads to the formation of pulmonary hypertension. However, this study has limitations. Firstly, this study is a pure bioassay, and basic experiments need to be established for verification. Secondly, the mechanism of P53 and/or telomere dysfunction in portal pulmonary hypertension remains to be further studied. In future work, we need to verify this experiment with in vitro and in vivo experiments. At the same time, the exploration of the molecular mechanism of the disease will help us to have a better understanding of the occurrence and development of the disease and can help prevent and treat the disease.

## 5. Conclusions

We summarized that the mechanism of pulmonary hypertension induced by portal hypertension may be related to telomere dysfunction and P53 overactivation. In addition, lipid metabolism and intestinal inflammation are also involved in this process.

## Data Availability

The raw data used to support the findings of this study are freely available from GEO datasets GSE77627, GSE113439, and GSE53408.

## Supplementary Materials

Supplementary Materials The gene includes the common downregulated genes between PH (including CPH,INCPH, and PH) and PAH analysed by DEG; the WGCNA(gene) includes the common genes between PH (including CPH,INCPH, and PH) and PAH analyzed by WGCNA; the appendix includes the specific method parameters, data, and website involved in this experiment.
