# Supplementary material for: Bioinformatics Analysis of Common Genetic and Molecular Traits and Association of Portal Hypertension with Pulmonary Hypertension
Source: J Healthc Eng. 2022 Oct 20;2022:9237701. doi: 10.1155/2022/9237701 (PMC9613398; doi:10.1155/2022/9237701)
Supplement: Supplementary Materials — The gene includes the common downregulated genes between PH (including CPH,INCPH, and PH) and PAH analysed by DEG; the WGCNA(gene) includes the common genes between PH (including CPH,INCPH, and PH) and PAH analyzed by WGCNA; the appendix includes the specific method parameters, data, and website involved in this experiment. [file 9237701.f1.zip › appendix.docx]

[appendix](javascript:;)

| **1.GEO DataSets** |  |
| --- | --- |
| GSE77627 | [GSE77627 - GEO DataSets - NCBI (nih.gov)](https://www.ncbi.nlm.nih.gov/gds/?term=GSE77627) |
| GSE113439 | [GSE113439 - GEO DataSets - NCBI (nih.gov)](https://www.ncbi.nlm.nih.gov/gds/?term=GSE77627) |
| GSE53408 | [GSE53408 - GEO DataSets - NCBI (nih.gov)](https://www.ncbi.nlm.nih.gov/gds/?term=GSE77627) |
| 2.WGCNA | 1.the top 25% genes(PAH) were selected according to their variance (CPH and PH took the top 5000 genes); 2.soft threshold beta was calculated using Pearson analysis;3.To cluster splitting, scale independence was set at 0.85,the soft thresholding power the soft threshold β in WGCNA analysis were PAH=5， LCPH=16, INCPH=12 and PH=20, the minimum module size was set at 30, and the deepSplit was set at 2. 4.Selected genes in the correlation module based on GS and MM values (ABS (GS)>.2& ABS (datKME$MM. Module)>.8). |
| 3.**DEG** | Using GEO2R of PubMed to analyze GSE77627 and GSE53408 databases online respectively, and then filtered the data in R language respectively, including: 1. Adjusted the logFC threshold. 2. Adjusted adj. P threshold. 3. Gene filtering without specific meaning (such as loC-beginning genes and Mir-/ MT- beginning genes). 4. Deleted data with both high and low expression. |
| 4.Human miRNA Disease Database (HMDD) | [HMDD v3.2 (cuilab.cn)](http://www.cuilab.cn/hmdd) |
| 5.miRpath | [DIANA TOOLS - mirPath (athena-innovation.gr)](http://diana.imis.athena-innovation.gr/DianaTools/index.php?r=mirpath/index) |
| 6 miRwalk | [Home - miRWalk (uni-heidelberg.de)](http://mirwalk.umm.uni-heidelberg.de/) |
| 7 Targetscan | [TargetScanHuman 8.0](https://www.targetscan.org/vert_80/) |
| 8 miRDB | [miRDB - MicroRNA Target Prediction Database](http://mirdb.org/) |
| 9.PanglaoDB | [PanglaoDB - A Single Cell Sequencing Resource For Gene Expression Data](https://panglaodb.se/) |
